# Emerging antiviral resistant strains of influenza A and the potential therapeutic targets within the viral ribonucleoprotein (vRNP) complex

**DOI:** 10.1186/1743-422X-11-167

**Published:** 2014-09-16

**Authors:** Alicia M Davis, Bryan J Chabolla, Laura L Newcomb

**Affiliations:** Department of Biology, California State University San Bernardino, 5500 University Parkway, San Bernardino, CA 92407 USA

**Keywords:** Influenza, Resistance, Antiviral, RdRP, NP

## Abstract

Emerging antiviral resistant strains of influenza A virus are greatly limiting the therapies available to stop aggressive infections. Genome changes that confer resistance to the two classes of approved antivirals have been identified in circulating influenza A viruses. It is only a matter of time before the currently approved influenza A antivirals are rendered ineffective, emphasizing the need for additional influenza antiviral therapies. This review highlights the current state of antiviral resistance in circulating and highly pathogenic influenza A viruses and explores potential antiviral targets within the proteins of the influenza A virus ribonucleoprotein (vRNP) complex, drawing attention to the viral protein activities and interactions that play an indispensable role in the influenza life cycle. Investigation of small molecule inhibition, accelerated by the use of crystal structures of vRNP proteins, has provided important information about viral protein domains and interactions, and has revealed many promising antiviral drug candidates discussed in this review.

## Background

Influenza A viruses are infectious agents spread through contact or aerosol droplets that result in a seasonal respiratory illness which can potentially lead to death. Highly transmissible influenza A viruses can reach pandemic proportions as seen in 1918, 1957, 1968, and 2009. The natural reservoir for influenza A viruses are aquatic birds, but many animals are susceptible to infection, including swine and humans. While humans are not readily infected with avian influenza viruses, in rare cases direct avian to human transmission has occurred. Swine are readily susceptible to avian, swine, and human influenza subtypes and provide a vessel for genome reassortment among different subtypes of the virus. The influenza A virus genome is made up of eight negative sense RNA segments (vRNA). Reassortment of genome segments between different influenza A subtypes can yield new influenza A subtypes that have potential to cause a human influenza pandemic. While the 1918 pandemic virus was found to be of wholly avian origin, the 1957 and 1968 pandemics contained segments of avian and human origin [1]. In this case it is unclear if swine or human were the vessel of reassortment; although swine are more readily infected with avian influenzas, thus providing more opportunity for reassortment, humans can be infected with avian influenza, and although rare, reassortment within a human host remains a possibility [1]. The 2009 influenza H1N1 pandemic contained segments of avian, swine, and human origin and was thus a triple reassortant that likely emerged from swine [2].

Influenza infection is typically prevented by annual vaccination. However, vaccines are not useful after infection or against emerging subtypes of influenza not targeted during vaccine production, as witnessed with the novel H1N1 pandemic in 2009. Therefore, antivirals that target specific proteins to inhibit virus replication are necessary to stave the spread of an emerging pandemic. The eight genome segments of influenza A virus encode 10 different coding mRNAs by way of alternate splicing [3] which result in over 12 proteins due to alternate translation [4–7]. Two viral segments code for the surface proteins HA and NA for which influenza subtypes are named. Three viral gene segments encode the RNA dependent RNA polymerase complex: PA, PB1, and PB2. At least two of these genome segments also encode alternate translation products including PB1-F2, PB1-N40, PA-X, PA-N155 and PA-N182 [4–7]. One segment encodes the nucleoprotein NP. Two segments, M and NS, undergo alternate splicing to produce NS1, NS2 (NEP), M1 and M2 proteins. With so many viral protein interactions required in various stages of the influenza life cycle, there are numerous potential target sites for antiviral treatments. Current antivirals target the activities of M2 and NA, but resistance is emerging. This review catalogs the current state of influenza antiviral resistance and describes promising new molecules targeting proteins within the viral ribonucleoprotein (vRNP), the complex comprised of the viral RNA genome, the RNA-dependent RNA polymerase (RdRP), and nucleoprotein (NP).

## Review

### Current antivirals and resistance

The current antivirals approved by the FDA are, in order of their release, Symmetra (amantadine), Flumadine (rimantadine), Relenza (zanamivir), and Tamiflu (oseltamivir). Amantadine and rimantadine are adamantane derivatives that target and inhibit the M2 ion channel. The M2 ion channel is an integral membrane protein responsible for release of vRNPs during infection [8]. By binding to the M2 ion channel, amantadine and rimantadine inhibit vRNP release and thus viral replication [9, 10]. The antivirals zanamivir and oseltamivir are NA or neuraminidase inhibitors. NA activity is required to release new virions from infected cells [11]. These drugs inhibit virus release from infected host cells by binding to the active site of the NA protein [12, 13].

Resistance against both classes of influenza antiviral treatments has been documented. Resistance to M2 ion channel inhibitors occurs via a triple amino acid deletion at residues 28–31 or single amino acid substitutions in the transmembrane region spanning residues 26–31 of the M2 protein [14, 15]. 100% of H3N2 influenza A viruses circulating in 2009–2010 and 99.8% of 2009 pandemic H1N1 were resistant to adamantanes [16]. Many resistant H1N1 isolates encoded V27A substitution, while resistant H3N2 isolates were found to encode substitutions at L26F, V27A, A30T, S31N, or G34E [17]. Resistance to adamantanes in H7N9 has also been documented and is acquired by substitution S31N [18].

Resistance to NA inhibitors is less common as this class of inhibitors was developed later. For example, 98.9% of tested 2009 H1N1 viruses remained susceptible to oseltamivir, 100% of 2009 H1N1 viruses tested remained susceptible to zanamivir [19], 100% of influenza A H3N2 tested remained susceptible to both oseltamivir and zanamivir for the 2012–2013 season [20]. However, multiple single amino acid changes in NA alter susceptibility to the approved neuraminidase inhibitors. Residues V116, I117, E119, Q136, K150, D151, D199, I223, H275, and N295 were selected to monitor for changes that confer drug resistance or reduce efficacy of the antivirals [21]. Resistance evolves during treatment, via single amino acid substitutions including changes to amino acids mentioned above such as E119V, I223R [22, 23], and H275Y [24], but also R292K and N294S [25]. Pandemic H1N1 2009 isolates with a substitution at I223R were resistant to neuraminidase inhibitors in addition to M2 ion channel inhibitors as discussed above [23]. Thus, while NA inhibitors are currently still viable to combat most emerging influenza threats, it is only a matter of time before resistance takes hold as with the adamantanes, rendering both current antiviral therapies ineffective against an emerging influenza threat.

**Figure 1:**
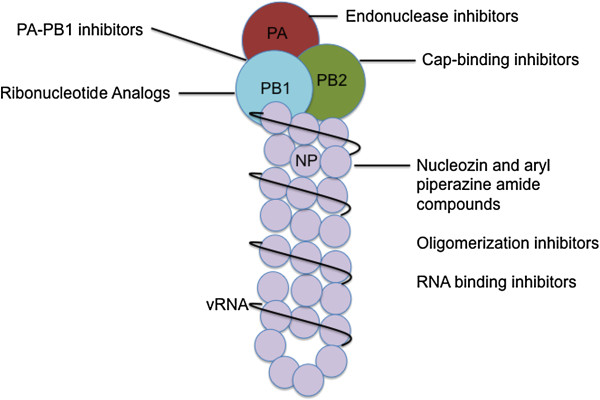
**Antiviral targets of viral ribonucleoprotein.** Anti-influenza candidates include small molecule inhibitors that disrupt critical functions or interactions within the vRNP. This figure provides a summary of the promising anti-influenza candidates discussed in this review.

Most worrisome is resistance reported among Highly Pathogenic Avian Influenza (HPAI) subtypes that could spur the next pandemic. Avian subtypes such as H5N1, H7N9, and H7N7 have all resulted in human infection. The H5N1 infections result in high morbidity at ~60%, while H7N9 and H7N7 have seen more variability in outcome of human infection [26]. Fortunately none of these subtypes have gained the ability to transmit readily from human to human, but unfortunately, these strains already have antiviral resistant isolates reported. For example, all H7N9 isolates tested were resistant to adamantanes via the S31N substitution in the M2 protein [27], while some H7N9 exhibit high resistance to oseltamivir, mid-resistance to peramivir, and low-resistance to zanamivir via the NA R292K substitution [28]. Also of grave concern are H5N1 isolates that encode M2 changes to confer resistance to adamantanes and NA changes that reduce susceptibility to neuraminidase inhibitors [29]. With many circulating antiviral resistant strains and the consequences of a looming virulent influenza pandemic, novel antiviral targets must be investigated so that new therapies can be developed before such a catastrophic event occurs. One promising novel viral target is the viral ribonucleoprotein or vRNP. Figure 1 depicts vRNP interactions and activities targeted by new anti-influenza candidates.

### vRNP: viral ribonucleoprotein

During infection eight vRNPs, containing the eight different vRNA genome segments, are released and imported into the nucleus to transcribe and replicate the vRNA. Transcription of the viral genome proceeds via a cellular capped-mRNA primer cleaved from host mRNA by the viral polymerase [30]. Unlike other RNA genome viruses, which typically replicate in the cytoplasm, influenza must enter the nucleus to steal nascent host capped mRNAs for use as primers in transcription. The polymerase subunit PA provides the endonuclease activity [31, 32], while PB2 houses the active site where the host pre-mRNA will bind [33]. Polymerization of the viral mRNA transcript proceeds via the PB1 subunit until the polyadenylation signal of repetitive U residues result in stuttering by the viral RdRP and yields the poly(A) tail, terminating transcription [34]. Transcripts of two influenza genome segments, M and NS, undergo alternative splicing to produce M1, M2, NS1 and NS2 (NEP); utilization of host nuclear splicing machinery is another reason for nuclear localization of influenza vRNPs.

Viral replication occurs *de novo*, without a primer [35]. In the host cell viral RNA replication occurs after translation of viral RdRP and NP proteins. Evidence suggests a model wherein the resident RdRP of the vRNP is responsible for transcription, while a soluble RdRP is responsible for replication from the vRNA template [36]. There are two primer-independent steps of viral replication. First the vRNA template is used to synthesize a full-length complementary (cRNA), which is then replicated to yield progeny vRNA that can be transcribed to mRNA or packaged into new virions during later stages of the viral replication cycle. NP is required for anti-termination at the poly U stretch to allow for replication of full length cRNA [37]. NP encapsidates both cRNA and vRNA replication products and is necessary for genome length functional cRNPs and vRNPs, respectively [38].

#### NP: nucleoprotein

**Figure 2:**
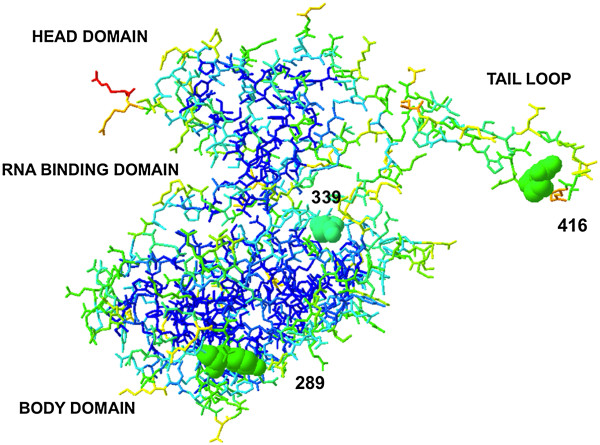
**Antiviral targets of nucleoprotein.** Analysis of NP monomer crystal structure extracted from NP trimer crystal structure “2IQH” [39] using Deep View-Swiss-PdbViewer 4.0. Residue color determined by accessibility. Greatest to least accessible as follows: red, orange, yellow, green, light blue, and dark blue. Residue 289 is the proposed site of interaction with the antiviral compound nucleozin [41]. An intermolecular salt bridge formed by residues 339 and 416 of NP is essential for oligomerization and can be disrupted by small molecule inhibitors [42].

The crystal structure of NP [39] (Figure 2) reveals two regions termed the body domain and the head domain, between which lies a deep groove comprised of positively charged basic amino acid residues that form ionic bonds with the negatively charged phosphate backbone of viral cRNA and vRNA. On the opposing side of the RNA binding groove lies a tail loop for oligomerization with other NP monomers. The tail loop of NP spans residues 402–428, and is critical to NP oligomerization and RNA binding, as shown through a tail loop deletion mutant that produced primarily monomeric NP unable to oligomerize and bind RNA [40]. Another crucial oligomerization interaction occurs through a salt bridge between residue 339 of one NP monomer and residue 416 of another NP monomer [39, 41, 42]. Mutational studies disrupting this salt bridge led to inhibition of viral RNA synthesis *in vitro*, further highlighting the importance of the interaction between NP molecules for vRNP function [42].

However, NP is more than a structural RNA binding protein. NP associates with viral proteins such as PB1 and PB2 [43], and M1 [44], in addition to several cellular factors [45]. Interaction between NP and the RdRP enhanced unprimed replication *in vitro*, suggesting the NP-RdRP interaction may regulate the switch from primer initiated transcription to unprimed replication [46]. Interaction of NP with the polymerase subunits was crudely mapped to regions within both the body and head domain of the NP crystal structure [43, 39]. NP-PB2 and NP-PB1 associations were confirmed through co-immunoprecipitation assays in the absence of other viral factors [43]. The NP-PB2 interaction was refined to residue 627 and 630 of PB2 and residue 150 of NP, with the strength of NP-PB2 interaction directly correlated with RdRP activity [47]. Residue R150 of NP is highly conserved and vital for normal viral RNA synthesis during influenza A WSN infection of MDCK cells [48]. As discussed below, PB2 residue 627 is a well-characterized host range determinant [49, 50]. Additional NP mutational analysis revealed alanine substitutions within three residues of the head domain at 204, 207, and 208 disrupt interaction with the viral polymerase and inhibit viral RNA synthesis [51]. Cryo-EM and cryo-electron tomography data of native vRNPs in packaged virions revealed two different conformations of polymerase interaction with NP within the vRNP [52]. Together, the evidence points to multiple residues on NP that participate in NP-RdRP interactions. The protein sequence homology of NP is a staggering ninety percent among influenza A isolates [53], with the described interaction domains exhibiting even greater homology, highlighting the potential of disrupting NP interactions as an effective antiviral target.

#### RdRP: RNA dependent RNA polymerase

The influenza A virus RNA dependent RNA polymerase is a heterotrimer comprised of PA, PB2, and PB1.

#### PA

While PA is required for both viral transcription and replication, the major role attributed to PA during influenza infection is the endonuclease activity needed to steal capped primers for viral transcription initiation in the nucleus of a host cell [30–32, 54]. Several highly conserved endonuclease active sites span the N-terminal 209 residues of PA [32]. Alanine screening of amino acids 102 through 134 revealed residues necessary for endonuclease activity [55]. Substitutions D108A and K134A individually inhibited both endonuclease activity and transcription *in vitro* [31, 55]. PA also makes contacts with both vRNA and cRNA promoters between residues 100-180, though the precise residues involved are debated [55–57]. In addition, PA has documented protease activity with Ser 624 defined as the active site [55], though the purpose of this activity remains poorly understood. The essential endonuclease activity of PA is an excellent target for antivirals.

#### PB2

PB2 contains the cap-binding domain, which recognizes the capped structure on nascent host mRNAs to be cleaved by the endonuclease site of PA [33]. PB2 residues 318-483 comprise this domain and contain two aromatic amino acids at positions 363 and 404 necessary for cap-binding [58, 59]. The cap-binding domain of PB2 may also mediate interaction between PB1 and PB2, specifically a loop consisting of residues 421–427 of PB2, which is essential for cap-dependent transcription but not cap-binding, as determined by a deletion mutant [59]. Cap-binding and capped RNA primed transcription are essential activities for influenza that can be targeted by novel antivirals.

PB2 also makes contacts with both vRNA and cRNA promoters in an alpha helix rich region between residues 535-684, which form an RNA binding domain [50]. Located within this domain is residue 627, a well-characterized species and pathogenicity determinant for influenza A viruses [49, 50, 60–63]. Avian influenza A viruses encode PB2 with glutamic acid at residue 627, while human influenza A viruses encode lysine [49]. RNA binding ability of PB2 was linked to the amino acid encoded at 627, with increased RNA binding shown for PB2 proteins containing K627 [50]. Polymerase activity and interaction of PB2 with NP were also shown to be influenced by PB2 residue 627, in addition to residue 630 [47]. Characteristic avian PB2 residue E627 must also encode R630 for proper polymerase activity and co-immunoprecipitation with NP [47]. Characteristic human PB2 residue K627 requires G630 for proper polymerase activity and co-immunoprecipitation with NP [47]. Residue 627 of PB2 influences binding of NP and RNA, two interactions essential for viral RNA synthesis, making this region of PB2 a favorable target for new antivirals.

#### PB1

PB1 is the RNA polymerizing subunit of the RNA dependent RNA polymerase. Residues 1-83 and 494-757 of PB1 contribute to vRNA template interaction through *in vitro* analysis of PB1 deletion mutants [64]. PB1 interacts with PA through its N terminal domain and PB2 through its C terminal domain, thus forming the functional RNA dependent RNA polymerase [65–68]. RNA dependent RNA polymerase activity of RNA viruses represents a viral activity that can be targeted by antivirals.

The C-terminal domain of PB1 (678-757) and the N-terminal domain of PB2 (1-37) were defined as the regions responsible for PB1-PB2 interaction and were crystalized to facilitate further investigation [66, 69]. Crystal structure of this interaction reveals all contacts occur through helix 1 of PB2 (residues 1–12), which is essential for proper RNA polymerase activity [69]. The PB1-PB2 protein interface is of great interest as an antiviral target due to the conservation of these domains in both human and avian viruses [69].

**Figure 3:**
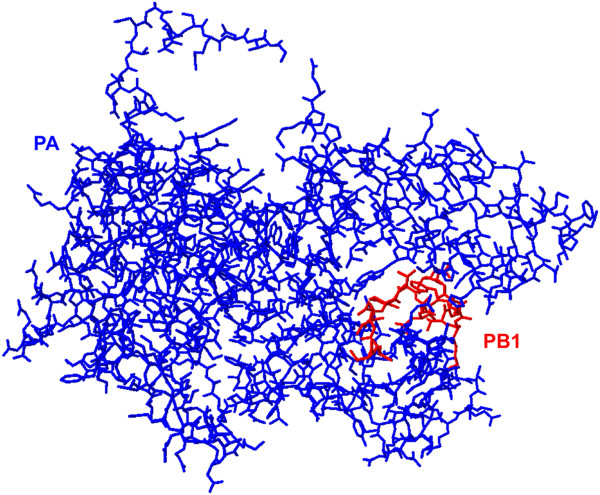
**PA-PB1 interaction site is an antiviral target.** Analysis of the PA_C_-PB1_N_ crystal structure “3CM8” [68] using Deep View-Swiss-PdbViewer 4.0. Residues 1–16 of PB1 (red) are shown in interaction with residues 258–716 of PA (blue). Many small molecule inhibitors of PA resemble the N terminus of PB1 and bind in the hydrophobic pocket blocking essential interactions between the polymerase subunits [68, 73, 95].

The N-terminus of PB1 (1–80) interacts with the C terminal region of PA consisting of residues 239–716 [66, 70]. Interaction surfaces of both PB1 and PA are highly conserved [68, 71, 72]. The crystal structure of this interaction reveals PB1 N terminal 25 residues occupy a C-terminal hydrophobic groove of PA [68] (Figure 3). The C-terminal domain of PA has been referred to as a “dragon’s head” that holds the N-terminus of PB1 in its “jaws” [68]. A peptide analog of the N-terminal 25 amino acids of PB1 blocks formation of the RNA dependent RNA polymerase complex resulting in no viral replication [68, 73]. These studies demonstrate the critical interaction between PB1 and PA in the formation of the RNA dependent RNA polymerase heterotrimer vital for viral RNA synthesis, making this interaction a potential target for novel antivirals.

### New antivirals targeting influenza vRNP

The critical roles of the influenza vRNP for viral RNA synthesis make activities of the vRNP, such as cap-snatching and RNA polymerization, excellent antiviral targets. A recently discovered nucleotide analog preferentially utilized by viral RNA dependent RNA polymerases including influenza vRNP, is under study as a promising antiviral therapy targeting the activity of viral RNA dependent RNA polymerases [74]. Further, the multiple essential interactions of the vRNP, such as with each other to form the RNA dependent RNA polymerase heterodimer, with host capped mRNAs to obtain primers for viral transcription, and with NP to regulate and enhance RNA replication, coupled with high conservation of these domains among influenza subtypes, make the proteins of the vRNP excellent targets for small molecule inhibitors with broad efficacy against multiple influenza A subtypes.

#### Ribonucleotide analogs

Favipiravir is a 6-fluoro-3-hydroxy-2-pyrazinecarboxamide molecule (also known as T-705) that upon phosphorylation becomes favipiravir-ribofuranosyl-5’-triphosphate (RTP) and inhibits many viral RNA dependent RNA polymerases [75]. Favipiravir is effective against influenza A, influenza B, influenza C, hantaviruses, flaviviruses, noroviruses, and most recently ebola viruses [74–76]. The T-705 RTP is erroneously interpreted as a purine nucleotide by the viral polymerase during RNA elongation [75, 77]. Once incorporated into the elongating viral RNA, the analog may hinder strand extension [77]. The antiviral activity of Favipiravir includes influenza A(H3N2), A(H1N1), A(H5N1), A(H7N9), and strains bearing resistance to both classes of the current FDA approved influenza antivirals [74, 75, 78].

The 50% inhibitory concentration (IC_50_) of favipiravir for influenza, determined by plaque reduction assay, was 0.013-0.48 μg/ml with no cytotoxic effect up to 1000 μg/ml [74]. Human DNA polymerase α, β, or γ with 1000 μM of favipiravir showed little sign of inhibition [79] and human RNA polymerase II had an IC_50_ of 905 μM of favipiravir [80]. Therefore, the IC_50_ of host polymerases is well over 2000 times greater then the IC_50_ for influenza vRNP, making favipiravir highly selective for influenza vRNP [75]. Favipiravir for influenza therapy has finished two Phase II clinical trials in the United States and one Phase III clinical trial in Japan [75]. Favipiravir is a favorable candidate for a broadly effective antiviral therapy targeting RNA viruses with RNA dependent RNA polymerases that preferentially incorporate favipiravir in RNA synthesis. It is not yet clear if or how quickly influenza vRNP will evolve resistance to this nucleotide analog, but based on HBV and approved nucleotide analog therapies [81], as with any antiviral therapy, if resistance is possible, it will eventually develop, stressing the need to consistently look for novel antiviral targets and therapies.

#### Small molecule inhibitors

Unlike nucleotide analog therapies, small molecule inhibitors work by interaction with a viral protein to block a function or interaction and inhibit viral replication. Crystal structure data provides much information to identify small molecules with potential to bind a domain of the target protein and inhibit essential functions or interactions. Small molecules that target conserved regions are likely to have the best efficacy against multiple subtypes. Further, conserved regions are less likely to tolerate mutation and evolve a viable virus with resistance to the small molecule.

#### Targeting NP

Nucleozin is a small molecule inhibitor of NP that works by promoting NP oligomerization, blocking nuclear entry through aggregation of NP molecules at the nuclear membrane [41] and inducing vRNP aggregation during cytoplasmic trafficking [82]. Molecular docking models identified two proposed nucleozin interaction sites at residue 289 and 309 of NP [41]. Both residues participate to stabilize the interaction; a tyrosine at residue 289 forms aromatic ring stacking with nucleozin, while an asparagine at residue 309 shares a hydrogen bond with nucleozin. Strains found to be nucleozin resistant encoded a histidine in place of a tyrosine at residue 289 of NP [41]. From the crystal structure of NP, residue 289 is relatively accessible to interact with nucleozin (Figure 2) [39, 41]. The 289H NP variant likely disrupts a critical point of interaction between NP and nucleozin. Sequence analysis of the NP gene of 3,881 influenza strains revealed Y289H substitution in 527 strains. Unfortunately the A(H1N1)pdm09 strain was shown to contain this single amino acid alteration, signifying it as a nucleozin resistant strain [41]. Nucleozin demonstrated a 50% effective concentration (EC_50_) of only 0.069 μM against influenza with 50% cytotoxic concentration greater than 250 μM [41]. Thus nucleozin still holds potential as a potent antiviral for strains of influenza housing a tyrosine at residue 289 [41]. In addition to nucleozin, there are several other aryl piperazine amide compounds discovered in parallel that target NP and inhibit virus replication in the same manner [41, 83–85]. Cianci et al. provide a detailed review into the efficacy of aryl piperazine amides, including nucleozin, as NP inhibitors [85]. Further analysis of these compounds could lead to the synthesis of an optimized aryl piperazine amide inhibitor of viral replication, even against the circulating nucleozin resistant strains of influenza.

The essential salt bridge for NP oligomerization at residues 339 and 416 was targeted for disruption by small molecule inhibitors (Figure 2) [42]. Peptides that mimicked the NP tail loop (residues 402–428) bound in the tail loop binding pocket and inhibited NP oligomerization, resulting in decreased viral replication by greater than fifty percent [42]. Random virtual screening of small molecules identified four compounds (termed # 3, 7, 12, and 23) that interrupt NP oligomerization and decrease viral replication [42]. The IC_50_ of compounds 3, 7, 12, and 23 ranged from 2.4 to 118 μM in cells challenged with influenza A/WSN/33 for nine hours at a multiplicity of infection (MOI) of 0.2 [42]. Although the targeted NP domain is highly conserved, more research will need to be carried out to ensure little to no development of resistance to the compounds. Importantly, cytotoxicity of these compounds will need to be assessed before they can be of use as antiviral therapies.

Mycalamide A is an antiviral and antitumor compound isolated from *Mycale* sponges but is unfortunately toxic to cells [86]. A photo-cross-linked chemical array identified analogs of mycalamide A that possess the ability to bind NP [87]. One compound inhibited viral replication up to 77% in a plaque assay using influenza virus (A/WSN/33) with no cytotoxic effect [87]. Binding affinity of the analogs was greatest within the N-terminal 110 amino acids of NP [87]. Although the mechanism of inhibition remains uncharacterized, the N-terminus of NP contains an important non-canonical nuclear localization signal (NLS) [88, 89] and interacts with host RNA processing factors UAP56 and URH49 [90, 91] proposed to enhance RNA replication [92]. More research is needed on these compounds to elucidate the mechanism of inhibition and determine if or how quickly influenza NP will evolve resistance.

Naproxen is an over the counter nonsteroidal anti-inflammatory drug that inhibits NP from associating with RNA in the RNA binding groove (Figure 2) [93]. From several molecular docking studies, residues Y148, Q149, R150, R355, R361, and F489 are believed to stabilize naproxen binding to NP [93]. Naproxen acts selectively upon monomeric NP and was shown to protect MDCK cells and mice from a viral challenge at an MOI of 10^-2^ - 10^-3^ or 50–2,000 PFU respectively, with little to no cytotoxic effects [93]. Subtypes of H1N1 and H3N2 were both susceptible to inhibition and treatment resulted in efficient protection from viral challenge with either subtype [93]. No escape mutant viruses were produced in response to 500 μM naproxen treatment in cells after six passages [93]. Within this experiment the mode of delivery for naproxen was intraperitoneal injection or intranasal treatment in mice that displayed an EC_50_ of 40 mg/kg [93]. Naproxen is currently used as an oral medication with a recommended dosage of 220 mg every 8 hours for pain. Naproxen is not currently used as an antiviral but could be optimized for antiviral use through further experimentation and improved drug design.

#### Targeting PA-PB1 interaction

Inhibitors of the PA-PB1 interaction are numerous [94]. Compound 1 was discovered through *in silico* screening [94] using the crystal structure of PA_C_-PB1_N_ (Figure 3) [68]. The hydrophobic pocket of PA houses compound 1 according to molecular docking studies, and inhibition of PA-PB1 interaction was demonstrated through ELISA and immunoprecipitation of PA [94]. Compound 1 inhibited RNA polymerase activity in a dose-dependent manner with an IC_50_ of about 18 μM assessed in a minireplicon assay using a firefly luciferase reporter gene, with no significant cytotoxic effect up to concentrations of 250–1000 μM [94]. Compound 1 inhibited viral replication in MDCK cells for several influenza A H1N1 and H3N2 strains, a swine-origin influenza virus, and an oseltamivir-resistant isolate, with IC_50_ ranging from 12.2 to 22.5 μM [94].

There are two FDA approved medications that in addition to their intended use also possess anti-IAV abilities due to their structural similarity with the N terminal domain of PB1 that interacts with the C-terminus of PA (Figure 3) [95]. Benzbromarone is approved to treat gout and hyperuricemia by promoting the excretion of uric acid. Diclazuril is most commonly used in veterinary medicine as an anti-coccidial. Although these drugs are FDA approved, appropriate drug dosages for use against viral challenges would need to be established before they could be employed for use against influenza A. Testing for the ability of viruses to gain resistance to these drugs must also be done. Benzbromarone and diclazuril could potentially be utilized if an influenza strain arises that possesses resistance to other antivirals available [95].

In addition, a compound derived from licorice, 18β-glycyrrhetinic acid (GHA), is a naturally occurring compound that exhibits some anti-IAV activity attributed to interaction with the C terminal domain of PA [96]. Molecular docking studies identified GHA as a ligand to PA_C_ [96]. GHA decreased polymerase activity 80%, as assessed by a primer extension assay for cRNA synthesis [96]. These preliminary findings need to be investigated further with more informative assays including *in vitro* PA-PB1 interaction inhibition study, *in vivo* polymerase activity assays in tissue culture, and *in vivo* infection in an animal model. There are many small molecules that mimic the N terminus of PB1 fit in the hydrophobic groove of PA to inhibit PA-PB1 interaction (Figure 3) and should be studied further for their potential as an anti-influenza A treatment.

#### Targeting PA endonuclease

Fullerene (C_60_) is a spherical molecule of sixty carbon atoms that exhibits anti-influenza activity [97]. Full length PA and an isolated PA endonuclease domain were tested in an *in vitro* endonuclease assay in the presence of fullerene derivatives. Seven fullerene derivatives were able to inhibit the endonuclease ability of the full length PA and isolated endonuclease domain [97]. Docking simulations reveal the fullerene skeleton fits nicely into the endonuclease domain active pocket [97]. MDCK cells were infected with influenza A H1N1 or H3N2 mixed with 0 to 100 μM fullerene derivative and immunostained for NP at 24 hours post infection to reveal significantly less NP in cells infected with virus pre-incubated with fullerene compared to a DMSO control [97]. Twelve derivatives of fullerene were tested and resulted in varying efficacy against influenza but showed no cytotoxic effect up to 100 μM [97]. More investigation into the activity and expression of viral proteins in response to fullerene needs to be conducted. Importantly, treatment applied post-infection needs to be investigated. This novel compound exemplifies yet another possible antiviral target within the vRNP.

#### Targeting PB2 cap-binding domain

Compounds that mimic the 7-methylguanosine moiety of the 5’ cap of mRNAs may serve as transcriptional inhibitors. Docking studies of the cap-binding domain of PB2 revealed 7-alkylguanine derivatives as potential inhibitory compounds [98]. Several compounds bind the cap-binding domain of PB2 with greater affinity than a biotinylated cap analogue [98]. These compounds should be further studied and optimized for anti-influenza activity.

## Conclusion

Influenza A virus continues to remain a human menace, in terms of both human health and global economic costs. The wide host range of influenza A virus, coupled with a lack of proofreading activity within the viral RNA dependent RNA polymerases, and a segmented RNA genome allowing for segment reassortment, provide influenza A virus with the ability to evolve rapidly. While yearly vaccination is protective against the strains and subtypes predicted to be circulating and represented during vaccine production, vaccination will not protect against an unseen, emerging subtype of the virus. Antivirals are the first line of defense for an emerging pandemic and resistance to current antivirals is already circulating within influenza A viruses, hastening the resolve to identify new antiviral therapies. The viral ribonucleoprotein (vRNP) is essential for viral replication, making it an ideal target for antivirals. Essential activities of the vRNP include cap-snatching activity required for viral mRNA transcription and RNA polymerase activity required for viral mRNA transcription and RNA replication. The interactions required to form functional vRNPs with these essential activities comprise the most highly conserved protein domains within influenza A subtypes. These interaction domains represent ideal targets for small molecule inhibitors, as these domains are less likely to tolerate mutations. Combination drug therapy is also a potential means to challenge emerging antiviral resistance. The vRNP provides multiple viral protein targets to reduce selection pressures and emergence of resistant strains. However, if mutations conferring antiviral resistance are tolerated, history dictates these mutations will be selected by use of the antiviral and propagate in circulating influenza A viruses. This means the search for new influenza A antiviral inhibitors should be ongoing until a universal vaccine is achieved.

## Authors’ information

AMD is a master’s graduate student at CSUSB conducting MS thesis research focused on the role of influenza A virus NP for viral RNA synthesis and gene expression in the laboratory of LLN. BJC is an undergraduate at CSUSB. LLN is Associate Professor of Biology at CSUSB and PI for influenza A research projects in the laboratory.
